# Free-standing conductive hydrogel electrode for potentiometric glucose sensing†

**DOI:** 10.1039/d1ra08956k

**Published:** 2022-02-14

**Authors:** Shogo Himori, Toshiya Sakata

**Affiliations:** Department of Materials Engineering, School of Engineering, The University of Tokyo 7-3-1 Hongo, Bunkyo-ku Tokyo 113-8656 Japan sakata@biofet.t.u-tokyo.ac.jp

## Abstract

Flexible conductive polymer hydrogels are attracting attention as an electrode material. Electrochemical biosensors with conductive polymer hydrogels have been developed because they have some advantages such as biocompatibility, high conductivity, 3D nanostructure, solvated surface, and enlarged interface. Conductive polymer hydrogels bearing receptor molecules such as enzymes in its 3D nanostructure enable the detection of target analytes with high sensitivity. However, because such hydrogels are fragile, they cannot stand on their own and a supporting substrate is required to fabricate them. This means that the loss of mechanical toughness is detrimental for their application to flexible biosensors. In this study, we have proposed a free-standing conductive hydrogel electrode with no coating on a substrate, which is composed of polyaniline with phenyl boronic acid including polyvinyl alcohol, for potentiometric glucose sensing. In addition, its electrical responsivity to glucose has been confirmed by investigating its mechanical properties at various glucose concentrations, considering the hydrogel compositions.

## Abstract 2

A free-standing conductive hydrogel electrode with no coating on a substrate is proposed for potentiometric glucose sensing.

## Graphical abstract

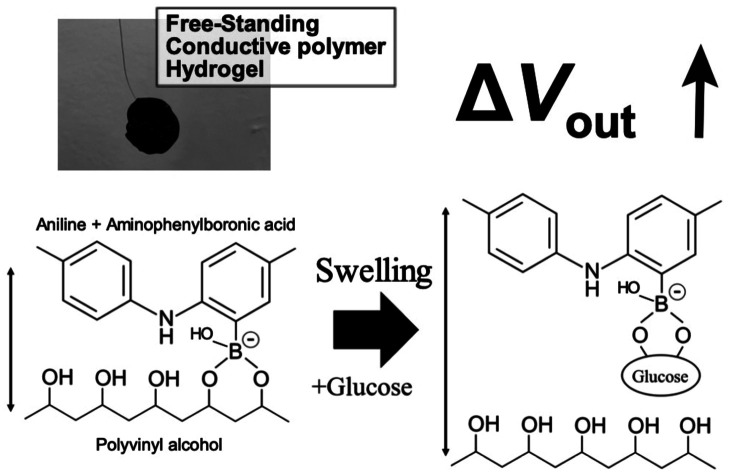

## 1. Introduction

Electrochemical biosensors are attractive tools for measuring biomarkers in humans for medical diagnosis. Electrochemical methods are conceptually based on a potentiometric or amperometric measurement principle. Potentiometric biosensors such as field-effect transistors (FETs) enable the direct detection of biomolecular charges based on biomolecular recognition events without redox reactions, regardless of their molecular sizes; therefore, such potentiometric biosensors are suitable for the direct detection of small biomolecules.^1,2^ As electrode materials, metal, carbon, oxide films, and so forth are often used.^3–6^ However, these electrode materials have a problematic issue of flexibility in the human body owing to their stiffness. Therefore, flexible conductive polymer hydrogels are attracting attention as an alternative electrode material.^7,8^ Moreover, conductive hydrogels have recently been developed for energy^9,10^ and biomedical applications.^11,12^ Electrochemical biosensors with conductive polymer hydrogels have been developed because they have some advantages such as biocompatibility, high conductivity, 3D nanostructure, solvated surface, and enlarged interface.^13^ Conductive polymer hydrogels bearing enzymes in its 3D nanostructure enable the detection of target analytes with high sensitivity.^14,15^ However, because such hydrogels are fragile, they cannot stand on their own and a supporting substrate is required to fabricate them. This means that the loss of mechanical toughness is detrimental for their application to flexible biosensors. In addition, the supporting substrate interferes with the utilization of hydrogels with their flexibility maintained and is a hurdle for the miniaturization of devices. Moreover, the contact between the hydrogel and the supporting electrode complicates the fabrication process. This is why the interface may be easily broken by some mechanical stimulations. Thus, mechanical toughness is necessary for the long-term stability toward a practical usage of flexible conductive polymer hydrogels. In this context, a free-standing conductive polymer hydrogel (FSC hydrogel) with high mechanical toughness can be applied to electrochemical biosensors.

**Fig. 1:**
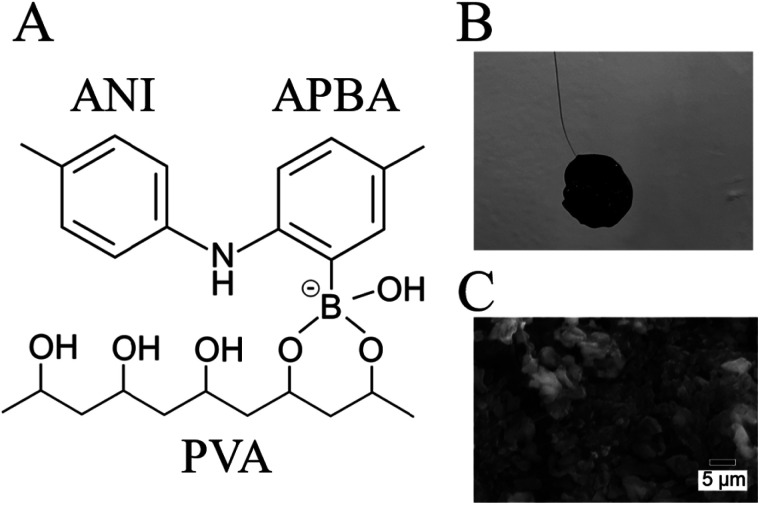
(A) Schematic of molecular structure in FSC hydrogel. (B) Photograph of FSC hydrogel with *ϕ*12 mm circular shape connected to Pt wire. (C) SEM image of FSC hydrogel. The hydrogel with the PVA ratio of 2 was observed.

Recently, FSC hydrogels have been developed for supercapacitors.^16–19^ One of the synthetic routes for the FSC hydrogels is based on *in situ* polymerization of monomers in an insulative matrix polymer.^17^ In particular, polyaniline (PANI), which is used as a conductive polymer, shows the expected electrochemical properties in polyvinyl alcohol (PVA) as an insulative matrix.^19^ In this synthetic route, amino phenylboronic acid (APBA) is copolymerized with ANI [P(ANI-APBA)] and used as a cross-linker with the PVA matrix on the basis of the specific binding between PBA and diol molecules such as PVA (Fig. 1A). The mechanical properties of the P(ANI-APBA)-PVA-based FSC hydrogels can be controlled by adjusting the composition ratio of PVA to copolymerized (ANI + APBA) because they cannot stand on their own. In particular, we pay attention to PBA as a recognition site of small biomolecules such as glucose and dopamine, which have diol groups in their chemical structures.^20,21^ It means the PBA–biomolecule binding, which induces molecular charges,^21^ contributes to a specific biomolecular recognition for next-generation electrochemical biosensors in an enzyme-free manner.^1^ On the other hand, the hydrogel-based biosensors with PBA have been recently developed for glucose detection in optical sensors.^22–27^ In these systems, the specific binding of glucose to PBA increases the density of negative charges and then induces repulsive force in the hydrogels; as a result, the hydrogels swell with increasing glucose concentration, resulting in changes in the optical properties of hydrogels. Thus, P(ANI-APBA)-PVA-based FSC hydrogels may enable the electrochemical detection of glucose as well.

In this study, an FSC hydrogel electrode was fabricated from ANI, APBA, and PVA in a one-step facile process, and its electrochemical responsivity toward glucose was investigated using a FET potential measurement system. In addition, its mechanical properties were analyzed by atomic force microscopy (AFM) to investigate their correlation with its electrochemical properties for glucose detection. This study can contribute to the utilization of conductive hydrogel biosensors with high mechanical toughness.

## 2. Experimental

### 2.1 Chemicals

Ammonium persulfate (APS), hydrochloric acid (HCl), aminophenyl boronic acid (APBA), aniline (ANI), polyvinyl alcohol (PVA), and glucose were purchased from Wako Pure Chemical Industries, Ltd. Phosphate-buffered saline (1× PBS, pH 7.4) was purchased from Thermo Fisher Scientific Inc.

### 2.2 Hydrogel electrode fabrication

For the fabrication of free-standing conductive hydrogel (FSC hydrogel), two types of solution (A and B) were prepared.^19^ Solution A was prepared by mixing 0.2 mmol of APS and 100 μl of HCl. Solution B was prepared by mixing 10.5 μmol of APBA, 0.15 mmol of ANI, PVA, and 400 μl of HCl. To investigate the effect of PVA amount on the free-standing property, solution B with 0.24, 0.32, 0.48, or 0.64 mmol of PVA was prepared. 18.8 μl of solution A and 100 μl of solution B were mixed and allowed to react overnight at 0 °C. The molar ratio of APBA + ANI to APS was 1 : 1 and that of APBA + ANI to PVA was 1 : *x* (*x* = 1.5, 2, 3, 4). Unless otherwise noted, the ratio of APBA + ANI to PVA was fixed at 1 : 2. The reaction solution was sandwiched at the top and the bottom by 12 mm-diameter round cover glasses (Matsunami Glass Ind., Ltd.) to keep the gel shape uniform, and part of the 0.10 mm-diameter platinum wire (The Nilaco Corporation) was immersed in the reaction solution to connect the gel to the measurement device. After gelation, the gel was kept in PBS for further experiments.

### 2.3 Electrochemical measurement

An electrochemical analyzer (618E, CH Instruments) was used to investigate the electrochemical properties of gel electrodes. The Ag/AgCl electrode was immersed in saturated KCl solution as a reference electrode. Pt was used as a counter electrode. The gel electrode was connected to the analyzer through a Pt wire.

Specific capacitance was calculated using the following equation using the result of cyclic voltammetry.^28^1
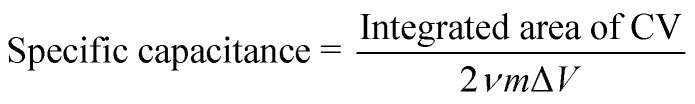
Here *ν* is the scan rate, *m* is the weight of the FSC hydrogel, and Δ*V* is the potential window.

For the FET potential measurement, the FSC hydrogel electrode was connected to the gate electrode of a silicon-based n-channel junction-type FET (K246, Toshiba), which is called an extended-gate FET, and a gate voltage was applied through the Ag/AgCl reference electrode (Fig. S1†). The surface potential at the FSC hydrogel gate electrode (*V*_out_) was measured in real time using a FET real-time monitoring system (PROVIGATE Inc.). In this study, the gate voltage (*V*_G_), drain voltage (*V*_D_), and drain–source current (*I*_DS_) were set to constant values, and the change in *V*_out_ (Δ*V*_out_) at the gate electrode was measured using a source follower circuit. In the electrical measurement, the reference electrode and the FSC hydrogel were put in 10 ml of PBS (Fig. S1A†). Δ*V*_out_ was monitored under the constant conditions of *I*_DS_ = 700 μA and *V*_G_ = 0 V. After the surface potential stabilized, small biomolecules were gradually titrated from a low concentration. To suppress the spike signals induced upon the addition of the sample, the introduced volume was 1/10 of the total volume of the measurement solution.

### 2.4 Force curve measurement by AFM

Force curve analysis was performed by atomic force microscopy (AFM; 5500 AFM, Keysight). The spring constant of the cantilever was 0.06 N m^−1^ and the radius of curvature was 20 nm with NP-O10 (Veeco) made of Si_3_N_4_. The FSC hydrogel was analyzed in the liquid cell with PBS. The force curve was taken from 4 × 4 points in 1 μm^2^ of each FSC hydrogel sample and used for calculation after omitting inconsistent curves. Each hydrogel sample was measured after immersing into each glucose solution for over 30 min for glucose to be absorbed into the hydrogel sufficiently.

## 3. Results and discussion

**Fig. 2:**
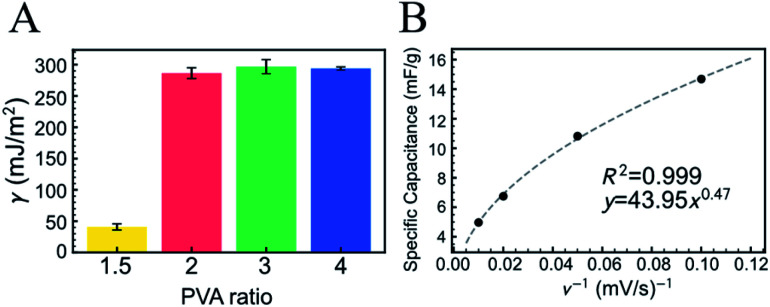
(A) Surface free energy (γ) of FSC hydrogels with varying PVA ratios. The molar ratio of PVA to copolymerized (ANI + APBA) was 1.5, 2, 3, or 4. γ was calculated using eqn (2) for the retraction force curve. The error bar shows the standard error of the mean and the measurement points were over 9. (B) Specific capacitance of FSC hydrogel for scan rate (*ν*) of cyclic voltammetry in PBS. The hydrogel with the PVA ratio of 2 was used.

The P(ANI-APBA)-PVA-based FSC hydrogel was formed with a 12 mm-diameter cylinder shape (Fig. 1B). The gel surface appeared flat, but the porous structure was observed by scanning electron microscopy (SEM) (Fig. 1C). Considering the adhesiveness of PVA in the P(ANI-APBA)-PVA-based FSC hydrogel, it was connected to the platinum wire as a gate electrode by physical bonding. The chemical composition and structure of the hydrogel were analyzed by attenuated total reflection Fourier transform infrared spectroscopy (ATR-FTIR). From the FTIR spectra (Fig. S2†), the characteristic peaks of PANI appeared at 1540 and 1500 cm^−1^, as derived from the vibrations of the quinoid and benzenoid rings, respectively.^29^ In addition, the peaks at 2920 and 1020 cm^−1^ were derived from the vibrations of C–H and C–O of PVA, respectively,^30^ and the peak of 690 cm^−1^ was attributed to the O–B–O bending of APBA.^31^ To investigate its free-standing property, force curve measurement was conducted by AFM. In the force curve analysis, the force is recorded when a cantilever is contacted with or retracted from the hydrogel.^32^ In the approaching curve, the cantilever pushes the hydrogel, resulting in the depression of the hydrogel as the force increases. On the other hand, in the retraction curve, the adhesion of the cantilever to the hydrogel caused the hydrogel to rise after the depression returned, and a negative force was observed (Fig. S3†). The maximum adhesion force (*F*_ad_) in the retraction force curve was calculated using the following eqn (2) based on the Johnson–Kendall–Roberts model:^32^2
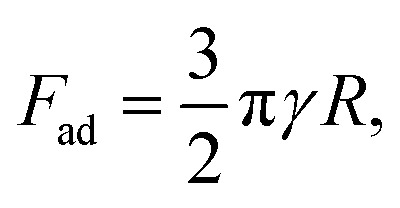
where *R* is the radius of curvature and *γ* is the surface free energy. According to eqn (2), the surface free energy was analyzed from the force curve measurement depending on the PVA amount, as shown in Fig. 2A. When the composition ratio of PVA (PVA ratio) to copolymerized (ANI + APBA) was 1.5 (see Experimental section), the surface free energy (around 40 mJ m^−2^) was similar to those of the PVA film (37 mJ m^−2^) and the PANI film (27–44 mJ m^−2^) reported previously.^33–35^ This means that when the PVA ratio was 1.5 or less, the cross-links were not sufficiently formed between PVA and APBA, that is, PVA and copolymerized (ANI + APBA) independently formed the polymer films instead of a well cross-linked hydrogel. On the other hand, the surface free energy increased to around 300 mJ m^−2^ when the PVA ratio was 2 or more. In our actual handling, the hydrogel with the PVA ratio of 1.5 was relatively fragile, whereas those with the ratio of 2 or more were sufficiently self-organized. Moreover, the aggregation of polymers should contribute to the increase in the surface free energy. Therefore, the introduction of PVA with the ratio of 2 or more induced the formation of more cross-links in the hydrogel, which resulted in its being free-standing. This result also indicates that the surface free energy can be an indicator for evaluating free-standing hydrogels.

The electrochemical property of the P(ANI-APBA)-PVA-based FSC hydrogel was examined by cyclic voltammetry (CV) in PBS (Fig. 2B and S4†). The specific capacitance of the P(ANI-APBA)-PVA-based FSC hydrogel with the PVA ratio of 2 was calculated using eqn (1) from the CV diagram (Fig. S4†) and analyzed for the sweep rate, as shown in Fig. 2B.^28^ The specific capacitance changed, depending on the reciprocal of the scan rate (*ν*^−1^), when other factors such as the mass and resistance of the electrode were constant. However, the specific capacitance was significantly smaller at a higher scan rate than at a lower one. This is a specific characteristic of porous materials owing to the change in the reactive area.^36,37^ At a higher scan rate, few ions in the measurement solution diffused into the electrode matrix; therefore, only the outer surface of P(ANI-APBA)-PVA-based FSC hydrogel electrochemically reacted with ions, resulting in the smaller specific capacitance. On the other hand, the lower scan rate contributed to the generation of greater electrical doping inside the porus hydrogel; therefore, the conductivity of the hydrogel increased, that is, the specific capacitance increased. Thus, the scan rate dependence of specific capacitance was useful for evaluating the porosity of FSC hydrogels.

**Fig. 3:**
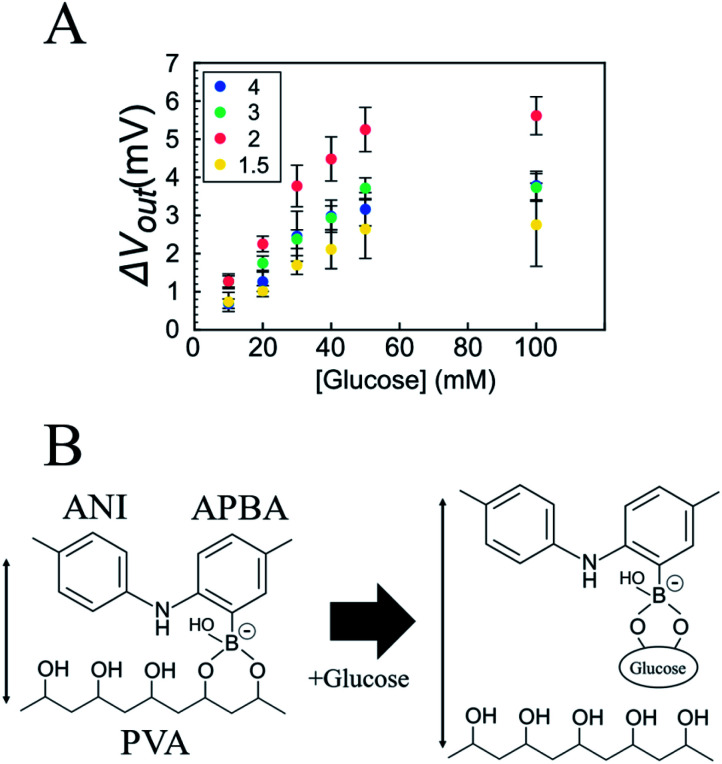
(A) Change in surface potential (Δ*V*_out_) of P(ANI-APBA)-PVA-based FSC hydrogel gate electrode with various PVA ratios with change in glucose concentration. The molar ratio of PVA to copolymerized (ANI + APBA) was 1.5, 2, 3, or 4. The error bar shows the standard error of the mean and the sample number was 3. (B) Schematic of glucose-induced swelling of P(ANI-APBA)-PVA-based FSC hydrogel.

The electrical responsivity of the P(ANI-APBA)-PVA-based FSC hydrogel electrode to glucose was investigated using the FET potential measurement system. The P(ANI-APBA)-PVA-based hydrogel was used as a gate electrode for an extended-gate FET (Fig. S1†). The change in the potential (Δ*V*_out_) of the P(ANI-APBA)-PVA-based FSC hydrogel electrode with the change in glucose concentration is shown in Fig. 3A. From the result, the potential increased with increasing glucose concentration, regardless of the PVA ratio. This may be because the capacitance of the FSC hydrogels increased upon adding glucose to them. Δ*V*_out_ was output with a source-follower circuit in this FET measurement system (see ESI†), on the basis of eqn (3) and (4):^38^3
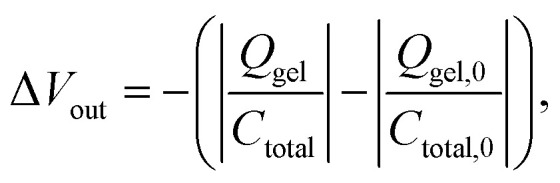
4
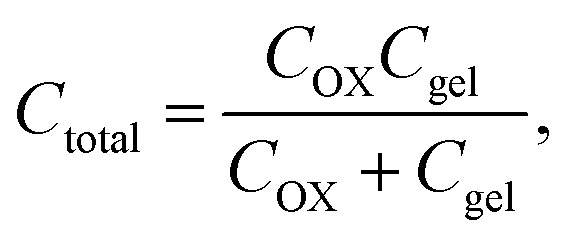
where *Q*_gel_ is the charge of the FSC hydrogel, and *C* is the capacitance of oxide (*C*_OX_) or the FSC hydrogel (*C*_gel_). These equations indicate that the change in the potential of the FSC hydrogel is calculated from the difference in *Q*/*C* from the initial state *Q*_0_/*C*_0_. Upon adding glucose to the P(ANI-APBA)-PVA-based FSC hydrogel in the measurement solution, PVA was replaced by glucose molecules because the binding constant of PBA to glucose (*K*_glucose_ = 110) is larger than that of PVA (*K*_PVA_ = 1.9).^27^ Therefore, the cross-links between APBA and PVA were released, and then the P(ANI-APBA)-PVA-based FSC hydrogel swelled (Fig. 3B).^26^ Here, the binding of diol molecules to unbound PBA decreases the acidity constant (p*K*a), resulting in the increase in the density of negative charges of PBA.^23,39^ However, since PBA had already been bound to PVA, the amount of molecular charges based on PBA should not have changed even when PVA was replaced by glucose. This means that Δ*V*_out_ is considered to have increased owing to the increase in the capacitance of the hydrogel caused by swelling, according to eqn (3) and (4). Furthermore, the P(ANI-APBA)-PVA-based FSC hydrogel with the PVA ratio of 2 showed the highest reactivity to glucose. The reason is broken down on the basis of two steps. Firstly, when the PVA ratio was over 2, the polymer density decreased with decreasing the amount of PVA. This is why the charge and discharge of the solution in the hydrogel contributed to greater glucose binding with PBA in the P(ANI-APBA)-PVA-based FSC hydrogel, resulting in the larger potential change for the P(ANI-APBA)-PVA-based FSC hydrogel with the PVA ratio of 2. Secondly, when the PVA ratio was 1.5, unbound PBA residues remained in the FSC hydrogel because the cross-links between PBA and PVA were not sufficient, as discussed for the surface free energy (Fig. 2A). After the binding of glucose to unbound PBA, the density of negative charges increased with decreasing p*K*a. This increase in the density of negative charges canceled the increase in the capacitance of the P(ANI-APBA)-PVA-based FSC hydrogel electrode for Δ*V*_out_ in eqn (3), and the glucose responsivity decreased. Therefore, it is suggested that both polymer density and cross-links in the P(ANI-APBA)-PVA-based FSC hydrogel should be controlled to increase the reactivity to glucose. Furthermore, the change in the specific capacitance with the change in glucose concentration was investigated using the P(ANI-APBA)-PVA-based FSC hydrogel electrode with the PVA ratio of 2 (Fig. S5†). The specific capacitance was calculated using eqn (1) from the CV diagram as described above (Fig. 2B). As shown in Fig. S5,† the specific capacitance response was particularly analyzed with increasing or decreasing glucose concentration, which showed the reversibility of response. In addition, the specific capacitance ratio at 100 mM glucose was larger than that at 10 mM glucose. This indicates that the P(ANI-APBA)-PVA-based FSC hydrogel swelled at the higher glucose concentration, which supported the observed Δ*V*_out_ with the change in the glucose concentration when using the extended-gate FET sensor (Fig. 3A).

**Fig. 4:**
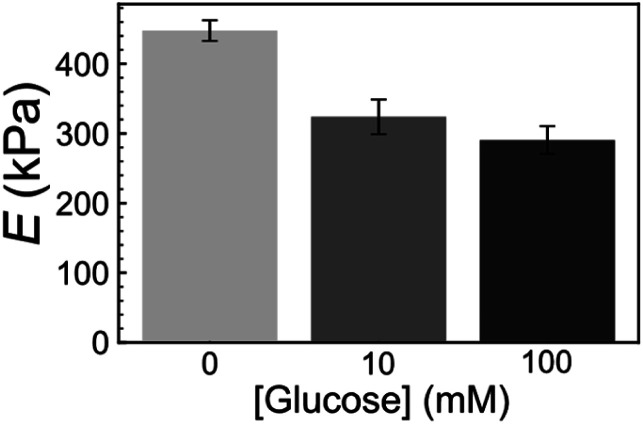
Young's modulus (*E*) of FSC hydrogel with PVA ratio of 2 at various glucose concentrations. *E* was calculated from eqn (5) using the approach force curve. The error bar shows the standard error of the mean and the measurement points were over 9.

Furthermore, we also examined the reactivity of P(ANI-APBA)-PVA-based FSC hydrogel with glucose on the basis of the mechanical measurement by AFM. The change in the Young's modulus (*E*) of the FSC hydrogel with the change in the glucose concentration was evaluated from the AFM force curve. On the basis of the Hertz model, *E* is obtained, according to eqn (5) from the approaching curve of AFM (Fig. S6†):^40^5
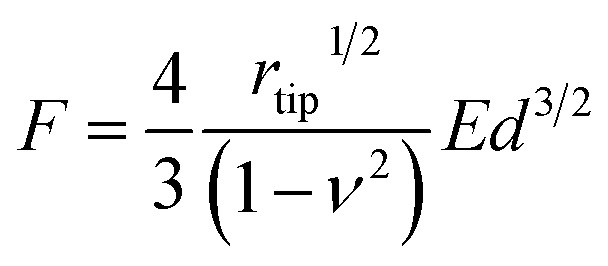
where *F* represents the force added to the FSC hydrogel, *d* the depth of deformation obtained in the approaching force curve, *r*_tip_ the radius of curvature, and *ν* the Poisson's ratio. Here, *r*_tip_ and *ν* were calculated to be 20 nm and 0.5, respectively, for the cantilever used in this study. As shown in Fig. 4, *E* decreased as the glucose concentration increased. This is because the P(ANI-APBA)-PVA-based FSC hydrogel swelled upon adding glucose to it owing to the replacement of PVA by glucose molecules for the cross-links with PBA. The decrease in *E*, which is based on the increase in the gel volume, has also been observed for another polymer in a previous study.^40^ Thus, the responsivity of the P(ANI-APBA)-PVA-based FSC hydrogel to glucose was also confirmed by the mechanical measurement.

## 4. Conclusions

In conclusion, we developed the P(ANI-APBA)-PVA-based FSC hydrogel electrode, which included the cross-links between PBA and PVA, and investigated its reactivity to glucose as a small biomolecule, focusing on both electrical and mechanical properties. In the electrical measurement using the extended-gate FET, the gate potential at the P(ANI-APBA)-PVA-based FSC hydrogel gate electrode increased with increasing glucose concentration, which was induced by increase in the capacitance of the FSC hydrogel owing to its swelling. In addition, the reactivity of the P(ANI-APBA)-PVA-based FSC hydrogel electrode to glucose was also clarified from its mechanical properties on the basis of the AFM force curve. The P(ANI-APBA)-PVA-based FSC hydrogel is suitable as a base electrode of electrochemical biosensors because it enables further chemical modifications of biorecognition units in/on its structure in the solution.

## Conflicts of interest

There are no conflicts to declare.

## Supplementary Material

RA-012-D1RA08956K-s001
