# Supplementary material for: Free-standing conductive hydrogel electrode for potentiometric glucose sensing
Source: RSC Adv. 2022 Feb 14;12(9):5369–73. doi: 10.1039/d1ra08956k (PMC8981371; doi:10.1039/d1ra08956k)
Supplement: RA-012-D1RA08956K-s001 [file RA-012-D1RA08956K-s001.pdf]

Electronic Supplementary Material (ESI) for RSC Advances.
This journal is © The Royal Society of Chemistry 2022

Electronic Supplementary Information

Free-standing conductive hydrogel electrode for potentiometric glucose sensing

Shogo Himori and Toshiya Sakata*
Department of Materials Engineering, School of Engineering, The University of Tokyo, 7-3-1 Hongo, Bunkyo-ku, Tokyo 113-8656, Japan
E-mail address: sakata@biofet.t.u-tokyo.ac.jp

1. Field-effect transistor (FET) potential measurement

The operation of a FET in the unsaturated region is described by the following equation S2.[S1,S2]

$$I_D = \mu C_{OX}\frac{W}{L}\left[(V_G - V_T)V_D - \frac{1}{2}{V_D}^2\right] \qquad (S1)$$

I_D is the drain current, μ is the electron mobility in the channel, C_{OX} is the gate oxide capacitance, W/L is the channel width to channel length ratio, V_D and V_G are the applied drain–source and gate–source voltages, respectively, and V_T is the threshold voltage, which is described by the following equation S2:

$$V_T = E_{ref} - \Psi_0 + \chi^{sol} - \frac{\varphi_{si}}{q} - \frac{Q_{it} + Q_f + Q_B}{C_{OX}} + 2\varphi_f\ , \qquad (S2)$$

Here, E_{ref} is the reference electrode potential relative to a vacuum, $-\Psi_0 + \chi^{sol}$ describes the interfacial potential at the electrolyte/silicon dioxide interface where the factor χ^{sol} is the surface dipole moment of the solution, which can be considered to be constant. φ_{si} is the electron work function of silicon, Q_{it}, Q_f, and Q_B are the charge of the interface traps, the fixed oxide charge, and the bulk depletion charge per unit area, respectively, and φ_f is the Fermi potential difference between the doped bulk silicon and the intrinsic silicon.

Taking into account for the capacitance and charge of the FSC hydrogels connected to the gate electrode, equation S2 is modified to

$$V_T = E_{ref} - \Psi_0 + \chi^{sol} - \frac{\varphi_{si}}{q} - \frac{Q_{it} + Q_f + Q_B + Q_{gel}}{C_{total}} + 2\varphi_f \qquad (S3)$$

with

$$C_{total} = \frac{C_{OX}C_{gel}}{C_{OX} + C_{gel}}\ , \qquad (S4)$$

where Q_{gel} is the charge of the FSC hydrogel and C_{total} is the combined capacitance of C_{OX} and that of the FSC hydrogel electrode (C_{gel}). The change in the interfacial potential ($\Delta\Psi_0$) at

the electrolyte/hydrogel gate electrode interface was constant because the ionic concentration was stabilized by the buffer solution. The quantities E_{ref}, $\frac{\varphi_{si}}{q}$, Q_{it}, Q_f, Q_B, and φ_f should remain unchanged by molecular binding to the hydrogel. In addition, ΔV_{out} at the gate was measured at constant I_D assuming the source follower circuit. Therefore, the detected ΔV_{out} was regarded as the change in V_{GS}, which equals $-|\Delta V_T|$ at constant I_D; then ΔV_{out} was calculated as follows:

$$\Delta V_{out} = -\left(\left|\frac{Q_{gel}}{C_{total}}\right| - \left|\frac{Q_{gel,0}}{C_{total,0}}\right|\right), \qquad (S5)$$

where $\frac{Q_{gel,0}}{C_{total,0}}$ is the initial state of $\frac{Q_{gel}}{C_{total}}$. These equations S5 and S4 correspond to equations 4 and 5 in the main text.

2. Additional data

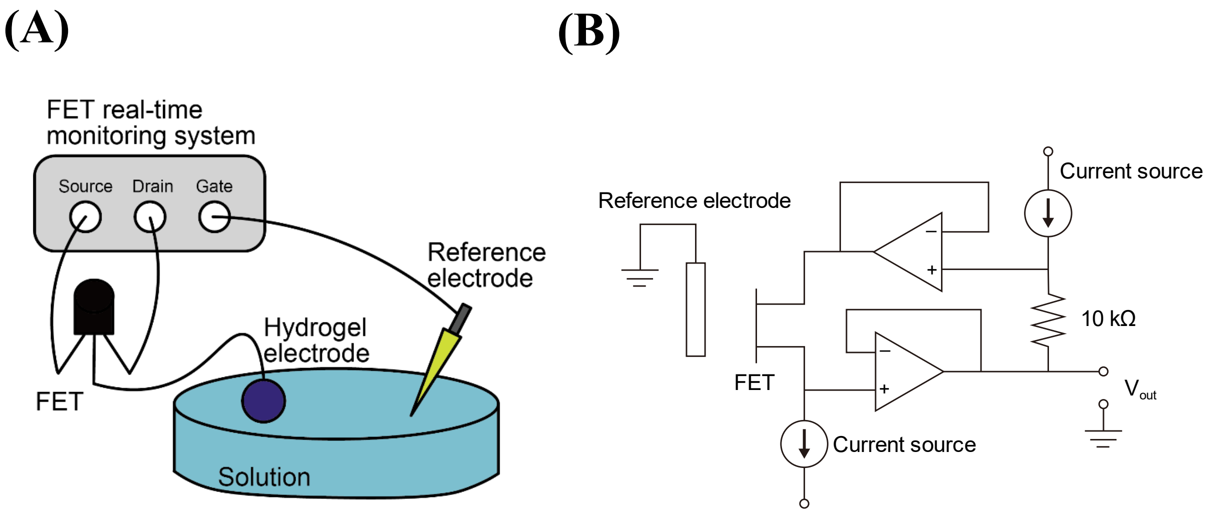

Figure S1 (A) Schematic of FET potential measurement system. **(B)** Electrical circuit. The change in surface potential (ΔV_{out}) at the gate electrode was measured at a constant I_D (700 μA) and V_G (0 V) using the source follower circuit.

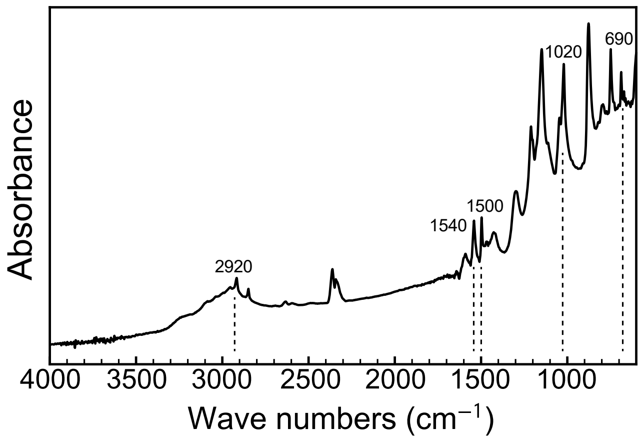

Figure S2 ATR-FTIR spectra of P(ANI-APBA)-PVA-based FSC hydrogel.

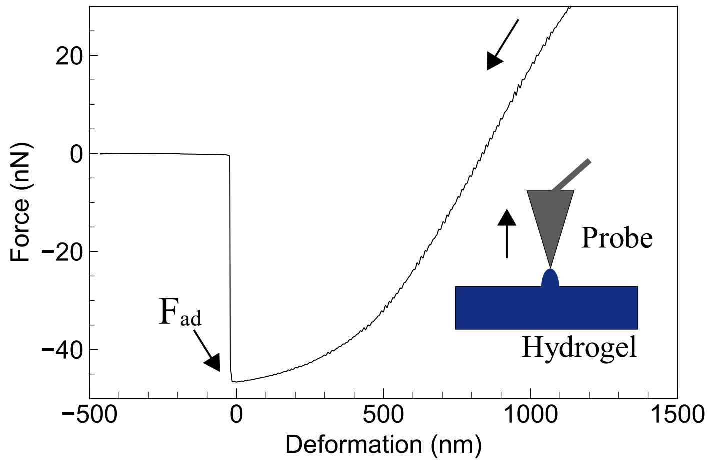

Figure S3 Retraction force curve on FSC hydrogels in PBS. The curve was analyzed using the Johnson–Kendall–Roberts model (equation 2).

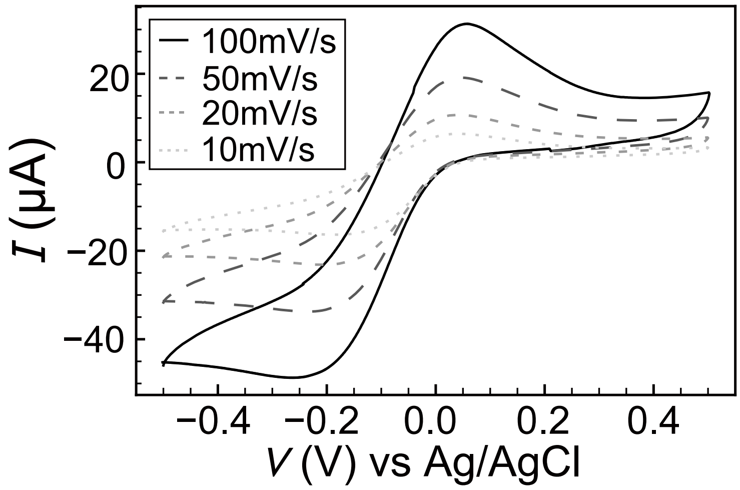

Figure S4 Cyclic voltammogram at scan rates of 10–100 mV/s. The hydrogel with the PVA ratio of 2 was used.

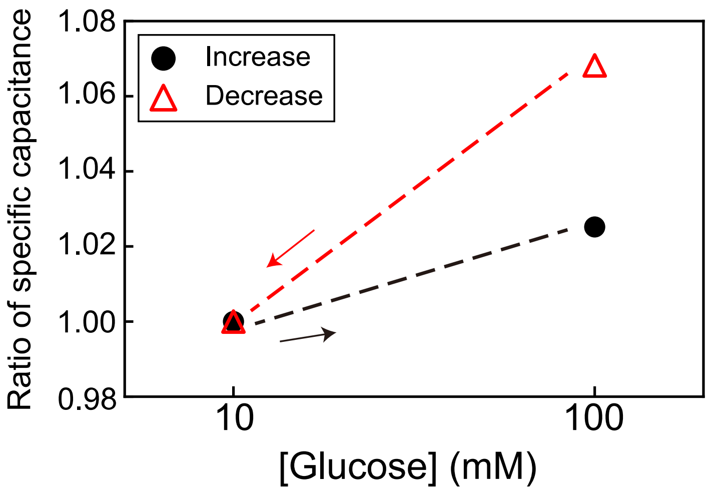

Figure S5 Ratio of specific capacitance depending on glucose concentration in PBS. Each specific capacitance was taken as a ratio to that of 10 mM glucose. Specific capacitance was calculated from the result of CV (−0.3–+0.9 V vs Ag/AgCl, 50 mV/s) with equation 1. Black circle, increasing concentration; red triangle, decreasing concentration.

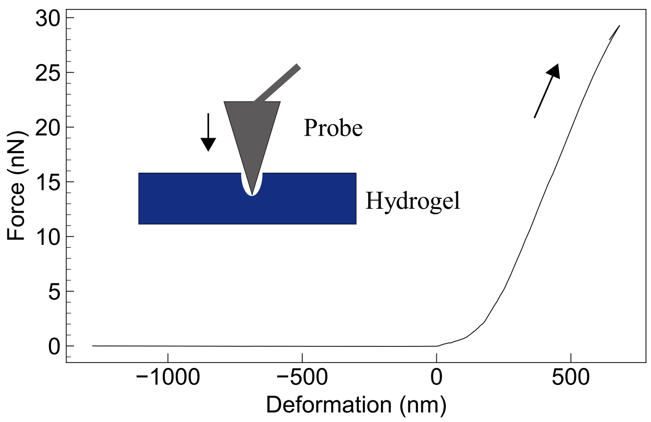

Figure S6 Approach force curve on FSC hydrogels in PBS. The curve was analyzed using the Hertz model (equation 5).

References

S1 T. Masuda, T. Kajisa, A. M. Akimoto, A. Fujita, K. Nagase, T. Okano, T. Sakata and R. Yoshida, *RSC Adv.*, 2017, **7**, 34517–34521.

S2 T. Kajisa, W. Li, T. Michinobu and T. Sakata, *Biosens. Bioelectron.*, 2018, **117**, 810–817.
